# Adherence to Vitamin Supplementation Recommendations in Youth Who Have Undergone Bariatric Surgery as Teenagers: a Mixed Methods Study

**DOI:** 10.1007/s11695-020-04880-y

**Published:** 2020-07-31

**Authors:** Anna Lena Brorsson, Karin Nordin, Kerstin Ekbom

**Affiliations:** 1 Department of Neurobiology, Care Sciences and Society, Karolinska Institutet, Alfred Nobels allé 23, SE-141 83 Huddinge, Sweden; 2 Division of Paediatrics, Department of Clinical Science, Intervention and Technology, Karolinska Institutet, SE-141 52 Huddinge, Sweden; 3 Division of Clinical Paediatrics, Department of Women’s and Children’s Health, Karolinska Institutet, Tomtebodavägen 18A, SE-171 77 Stockholm, Sweden

**Keywords:** Adolescent, Bariatric surgery, Gastric bypass, Nursing, Young adult

## Abstract

**Purpose:**

Adherence to vitamin supplementation recommendations after bariatric surgery is generally poor, which is associated with nutritional deficiencies. Patients’ own perspectives and reasons for poor adherence to vitamin supplementation recommendations have not yet been studied in depth. The purpose of this study was first to measure the accuracy of self-reported adherence to supplementation recommendations by using objective measures of vitamin D levels in blood and thereafter to explore perceptions of barriers and facilitators to participants’ adherence to supplementation recommendations.

**Material and Method:**

Participants were recruited from a prospective study investigating the outcome of bariatric surgery in adolescents with severe obesity. Mixed methods were used, including a quantitative part where vitamin D levels were analysed through 25(OH)D levels in blood and/or a questionnaire on adherence to supplementation programmes 5 years after surgery (*n* = 40) plus a qualitative part with semi-structured interviews (*n* = 20).

**Results:**

We found a convergence between self-reported adherence to vitamin supplementation and vitamin D 25(OH)D levels in blood indicating honestly in self-reported responses. The qualitative evaluations resulted in the categories awareness and personal capability and external factors. In the analysis, an overall theme emerged; capacity is crucial for adherence in youth who have undergone bariatric surgery.

**Conclusion:**

Bariatric surgery is a comprehensive procedure that requires lifelong treatment afterwards. There is coherence between what adolescents actually do and what they say they do. Capacity is crucial for adherence and social support has been shown to be important.

## Introduction

Bariatric surgery has been shown to be effective for long-term weight loss and as a treatment for obesity-related comorbidities, but it is associated with various micronutrient deficiencies [1]. Hypovitaminosis D is one of the most common deficiencies experienced after bariatric surgery, and the deficiency is multifactorial, with some factors related to obesity and others to nutrient malabsorption [2]. Little is known about nutritional deficiencies in adolescents, but nutritional risks have previously been described as higher among adolescents undergoing bariatric surgery than among adults. These differences may be explained by adults having better adherence to post-surgery supplementation [3–5]. Supplements are routinely recommended to minimize the possible risk of nutritional deficiencies [6], and daily supplementation of multivitamins including calcium and vitamin D is believed to be a long-term requirement for post-bariatric surgery [7]. However, there is some disagreement about supplementation due to the high variability in the response to vitamin D supplementation in patients who have undergone bariatric surgery. This may be explained by individual body fat levels and degrees of malabsorption [2].

Behaviour that dictates adherence to medication recommendations is complex, and since the 2003 World Health Organization’s report on adherence, little has improved with adherence levels, which are estimated to be 50% [8].

Barriers for adherence to supplementation recommendations after bariatric surgery have been reported before, and “difficult to remember” and “trouble taking all the tablets” were identified as two important barriers [9]. Some researchers have compared adherence with vitamin supplements in adolescent and adult bariatric populations [10, 11], and rates of vitamin adherence have been as low as 30% 6-month post-surgery in adolescents [10]. In adults, self-reported non-adherence was associated with lower vitamin levels [11]. In a recently published study, 38% of adolescents had low levels of vitamin D compared with 24% of adults, with the difference being explained by the younger patients’ lack of adherence to vitamin supplementation recommendations after surgery [4].

It has been suggested that adolescents adhere better to recommendations that have immediate consequences if they are not followed than those that have less obvious benefits or that are intrusive to their lifestyle [12]. However, rates of adherence in adolescents vary widely, and a lack of consistency in measurements may contribute to this variation [13]. Non-adherence to recommendations after bariatric surgery has been linked with poorer outcomes, and adherence may be a key to a successful outcome [14]. Preoperative predictors of adherence to dietary and physical activity recommendations have been studied, and this research has highlighted the importance of preoperative psychosocial preparatory work [15].

The long-term risks of bariatric surgery are not well identified, especially the skeletal risk where vitamin D deficiency may play a part in increased bone mineral density loss after bariatric surgery [16], and a detailed understanding of the long-term nutritional effects of the surgery in this age group is limited. Therefore, it is of clinical importance to acquire coherent knowledge on whether adolescents do what they say they do and barriers and facilitators to following prescribed recommendations, in order to be able to provide support to these individuals.

The purpose of this study was first to measure the accuracy of self-reported adherence to supplementation recommendations by using objective measures of vitamin D levels in blood and thereafter to explore perceptions on barriers and facilitators to adherence to supplementation recommendations.

## Material and Methods

In this study, mixed methods have been used consisting of a quantitative descriptive section and results from a qualitative inductive interview study that includes participants from the Adolescents Morbid Obesity Surgery (AMOS) study. A description of this study, including 2- and 5-year follow-up results on weight, metabolic risk markers, and quality of life indicators, have been previously reported [17].

### Participants and Setting

**Fig. 1:**
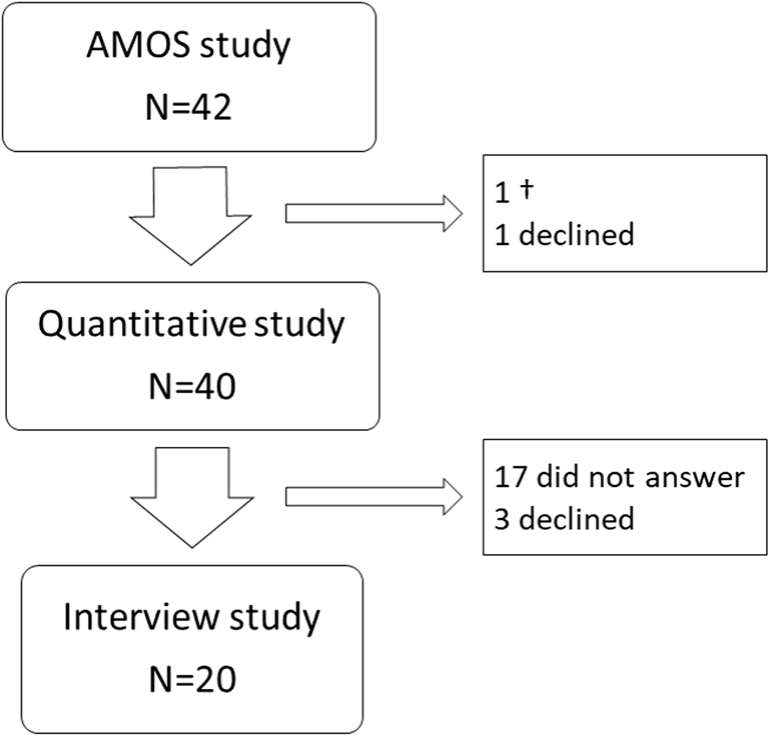
Flowchart of included participants

Participants were recruited from the Adolescent Morbid Obesity Surgery (AMOS) study, a prospective Swedish non-randomized multicentre study investigating the outcome of bariatric surgery in adolescents with severe obesity. All patients had surgery at the same centre (Gothenburg, Sweden) and were treated according to the same procedures before and after surgery [17]. For the quantitative study, all 42 AMOS participants based in the greater Stockholm area are included (Fig. 1). They were all asked to participate in the interview study, and 20 (49%) accepted, 4 (10%) declined, 17 (41%) did not answer, and one had passed away due to causes unrelated to the surgery.

According to international guidelines [18], the adolescents were prescribed daily multivitamin and mineral supplements after surgery, including 200 μg folic acid, vitamin B12 (cobalamin 1 mg/day), and calcium carbonate and vitamin D (1 g/800 IU per day) combination tablets. Every year during their regular follow-up visits, blood samples were taken for the analysis of metabolic parameters, minerals, and vitamins. Vitamin D status was measured as 25-hydroxyvitamin D (25(OH)D). The cost of the prescribed supplements is estimated to be 120 Euros annually.

The study was approved by the ethical board in Västra Götaland, Sweden (523-04), and informed consent was obtained from all participants.

### Data Collection

#### Quantitative Data

For the descriptive study, all AMOS patients from the Stockholm area with a laboratory analysis of 25(OH)D 5 years after surgery and/or who answered a question about adherence to supplementation were included (*n* = 40). The question asked was “Do you take your prescribed vitamin supplementation?”, which was created specifically for the study based on clinical experience. Demographic data including body mass index standard deviation score (BMI SDS) [19], weight loss/regain, vitamin D status including 25(OH)D levels in blood, and self-reported assessments of adherence were collected.

#### Qualitative Data

Based on the identified knowledge gap and clinical experience, two of the authors (KN and KE) created open-ended qualitative questions to be used in the interviews. Each question was followed up by probing questions with the aim of this leading to a dialogue [20, 21]. The follow-up questions were adapted for young adults; some of whom may still depend on caregivers. The interviews were carried out 5.75 to 9.75 years after participants had undergone bariatric surgery. One of the authors (KN), who is a registered nurse with extensive clinical experience of conversations with adolescents, conducted the interviews. The questions this analysis is based on were as follows: Can you describe how you followed the advice on how to eat after surgery? Can you describe how you followed your prescribed treatment regarding vitamin supplements? Do you have any suggestions for action that could improve adherence to dietary advice and the intake of vitamins?

Participants were given the opportunity to choose a convenient time for their interview. All interviews were conducted over the telephone and lasted for 30–60 min. All interviews were audio recorded and thereafter transcribed verbatim by one of the authors (KN).

### Data Analysis

#### Quantitative Data

Two different measurements of adherence to prescribed treatment were used.

1. Direct method: 25(OH)D levels in blood at least 5 years after surgery. The definition given by the US Institute of Medicine (IOM) was used for classifying the following vitamin D status groups [22]: adequate, 25(OH) levels > 50 nmol/l; insufficient, 25(OH) levels 30–50 nmol/l; and deficient, 25(OH) levels < 30 nmol/l.
2. Indirect method: all adolescents were asked if they adhered to prescribed treatment 5 years after surgery and were given three alternatives (yes/no/sometimes). For classifying the indirect method of adherence, yes was classified as good adherence, sometimes as poor adherence, and no as very poor adherence.

#### Qualitative Data

Qualitative content analysis inspired by Krippendorff was used to analyse the data [23]. The interview recordings were listened to and re-read several times. Based on the purpose of the study, units of meaning were identified and then condensed and labelled with codes by all the authors. Thereafter, the codes were discussed in the research group. Different codes were compared and divided into subcategories based on similarities and differences. The analysis was based on a manifest interpretation of the text. At the end of the analysis, a latent interpretation of the content could be made and an overall theme emerged [24].

### Statistics

Descriptive statistics were expressed as mean, min-max, number, and percentage. Frequencies were presented in cases and percentages. Fisher’s exact test was used to test the degree of association between categorical variables. We compared proportions by means of chi-square tests when expected values for any cells of contingency tables were five or higher, and we used Fisher’s exact test if otherwise. All tests were two-sided, and *p* values of < 0.05 were regarded as significant. Statistical analyses were performed using SPSS Statistics version 22 (IBM, Armonk, NY, USA, http://www.ibm.com).

## Results

### Quantitative

A total of 40 adolescents (31 females, 9 males), aged 14.9 to 18.3 years when they underwent bariatric surgery, were included in the analysis.

When adherence to vitamin D supplementation recommendations was measured using 25(OH)D levels, adequate vitamin D levels were found in 17 (43%) of the adolescents, twelve (30%) had insufficient vitamin D levels, and 11 (28%) had deficient vitamin D levels. When adherence was measured using the indirect method, 18 (46%) of the adolescents reported good adherence, 10 (28%) reported poor adherence, and 11 (26%) reported very poor adherence.

We found a significant association between self-reported adherence to vitamin D and vitamin D levels in blood (*p* = 0.02). In summary, the rates of agreement are found to be 15% false positive and 10% false negative (Table 1).

**Table 1:** Association between self-reported (SR) adherence to vitamin D and 25(OH)D levels in blood

25(OH)D levels	SR good	SR poor	SR very poor
Adequate	30%	7.5%	5%
Insufficient	7.5%	5%	5%
Deficient	7.5%	15%	17.5%

*SR* self-reported

In line with our results above, significantly higher 25(OH)D levels were found in the self-reported group answering “Yes I am taking the supplements” than in the groups answering “no” or “sometimes” (*p* = 0.008), while no differences were found when comparing weight regain/loss (data not shown).

### Qualitative Evaluation

**Fig. 2:**
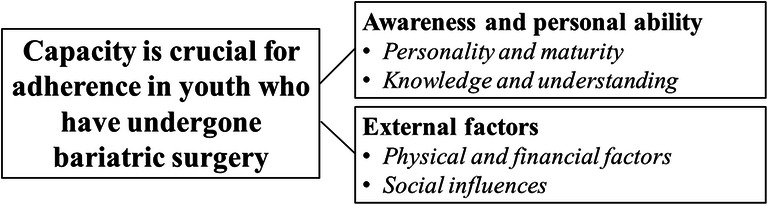
Description of the overarching theme, categories, and subcategories

The qualitative evaluation of barriers and facilitators for the adherence to prescribed supplementation in young adults who have undergone bariatric surgery resulted in two categories: awareness and personal capability and external factors. In the analysis, an overall theme emerged; capacity is crucial for adherence in youth who have undergone bariatric surgery (Fig. 2).

#### Awareness and Personal Capability

##### Personality and Maturity

Personality had a significant role in adherence to supplementation programmes. Some of the participants described that people with a personality that goes “all-in” for things decide to undergo surgery and thereby commit to taking supplements for the rest of their lives. An awareness of the importance of maturity was described, and taking more responsibility for self-management is a part of becoming an adult. It was described as being easier to follow dietary recommendations and vitamin supplementation advice when they were older. Some highlighted that it takes time for routines to be established. One participant described the importance of assessing maturity and readiness to change before bariatric surgery is performed, since this is more important than age.

“It depends on how mature you are as a person … I’m not saying that children shouldn’t get surgery, but I think that personality type and how you are as a person is important.”

Participants described various creative strategies they had developed to help them remember to take the supplements. It seems important to make it a routine in everyday life. Success factors include taking them at the same time each day and keeping them where they are visible. Furthermore, setting an alarm on a phone helped participants to remember to take them.

“I have the routine of taking them in the morning when I wake up… I have them in a pill-organizer and I have the pill-organizer visible, so I remember.”

Some participants openly admitted not taking the prescribed supplements, but they could not give any specific reasons why. They described themselves as sloppy because they forgot, but they stated that it was their own responsibility. Some adhered to the prescribed supplementation programme for periods of time, but after a while, their adherence faded. This was connected to difficulties in establishing long-term daily routines for self-management. One participant had even given up trying to remember to take the supplements. Several participants described lacking daily routines for taking the supplements.

“... I did well the first year but since then I have been sloppy; I take them for a period and then I forget about them again. Then I think I should take them again and then I do for a period and then I forget again.”

Similar patterns were present for sampling, where participants described that they were aware that they were supposed to control their vitamin levels, but they could not explain the reasons behind their lack of adherence.

The follow-up default for those who moved to another town was that they did not take responsibility for establishing contact with a new healthcare provider. This resulted in no follow-up visits being conducted, and no supplements being prescribed. Some waited until they experienced malaise or tiredness before contacting their healthcare provider.

“I have moved and started studying and haven’t had time to book an appointment with a doctor, it is a new healthcare center and so on.”

### Knowledge and Understanding

There seemed to be a certain amount of ignorance among participants regarding the importance of supplementation; they were not aware of any significant difference in their wellbeing when taking the supplements. The participants expressed some aversion―a distrust regarding supplementation and whether it had any effect. Some claimed that vitamins were unnecessary―changing eating habits and eating a lot of vegetables were perceived as a better approach because it was more natural than taking supplements. However, participants described discussing vitamin deficiency during medical visits. They suggested that regular reminders about the consequences of not taking supplements could be good for adherence.

“... you benefit from the vitamins, because you get a more balanced diet and get nutrients in the right way.”

One participant described the importance of information being provided in a gender- and age-appropriate manner. Some pointed out that frank information about the physical consequences that could be suffered if supplements are not taken would be beneficial.

“... to girls, you can say that if you don’t take the supplements it is very possible that you will get very weak nails and for some reason, nails are very important to girls … Or you will lose your hair and get much thinner hair.”

#### External Factors

##### Physical and Financial Factors

Several underlying causes are described as reasons behind insufficient adherence. One of the most prominent reasons was experiencing difficulties in swallowing tablets, which are described as large and many in number. In addition, they should be administered at different times, which made it difficult and sometimes resulted in the tablets being forgotten.

“... there’s a lot of them, several times a day – it’s difficult to combine them when there are so many.”

“It’s really important, they’ve always said that, but it’s hard to "get them down" and to remember them.”

Iron is one of the supplements described by the participants as causing gastrointestinal side effects. These were experienced as very troublesome and often led to them being skipped.

Another factor that emerged as important was personal finances. The cost of supplements and visits to healthcare providers were seen as expensive and experienced as time consuming.

“I have avoided buying them because they are so terribly expensive.”

### Social Influences

Several participants described how relatives have affected their adherence to supplementation recommendations. It could be a grandmother who got angry if prescribed supplements were not taken, a reminder from a partner, or support from parents or siblings. Those who had relatives who had also undergone bariatric surgery found this helpful.

” ... and then because mum and my sister have done the same surgery and take almost the same tablets, it has also helped. You think that they take them so maybe you should too…”

One participant described how a pregnancy made her realize the importance of following prescribed recommendations.

“Before, during, and after my pregnancy it has been easier because I have someone else to think about. Now I think I need them even more because I am breastfeeding.”

## Discussion

Our study was based on two different methods to describe adherence to supplementation recommendations after bariatric surgery to determine the convergence between self-reported adherence and vitamin D status and explore the reasons behind insufficient adherence.

The results in our study show convergence of self-reported adherence and levels of vitamin D. Two categories emerged in the analysis: awareness and personal capability and external factors. It was clear that it was crucial if you had insight into why the supplements were prescribed and for what reason. Most of the participants stated that they did not take their supplements because the tablets were large and there were so many. A clear facilitator was whether there were people or social circumstances that motivated them.

Some previous studies have reported blood levels of vitamin D as a proxy for adherence but did not report the relationship between blood analyses and self-reported adherence [13]. Our results indicate that self-reported adherence is concordant with blood analysis, indicating that the participants who stated that they followed the supplement recommendations answered truthfully.

Some studies have shown that self-reported adherence can be a predictor of clinical outcomes in patients with other diagnoses [25], while other studies have reported no correlation between self-reported adherence and objective measurements of adherence [10]. However, a high proportion of participants in our study, approximately 50%, were non-adherent patients, which is in line with a previous observational and intervention study, especially in adolescents as younger age groups are linked to lower adherence levels [7]. A previous study of adults reported adherence to vitamin D supplementation to be good just after surgery (90%) with regular follow-ups and nutritional advice, but this rate dropped to approximately 50% 1 year after surgery [26].

Our qualitative evaluation resulted in an overarching theme to describe facilitators and barriers: Capacity is crucial for adherence in youth who have undergone bariatric surgery.

Self-efficacy and social power have been described as central to adherence [27]; therefore, in order to understand our results, we have used a framework based on this concept [28, 29]. Self-efficacy is described as one’s feelings and thoughts about one’s capability [28]. Power in healthcare may be described as “the capacity to participate knowingly in change” and consists of four inseparable dimensions―awareness, choices, freedom to act intentionally, and involvement in creating change [29, p 48–49]. In this context, social power, including referent power, may be relevant, which is a person with the ability to be a frame of reference, for example, a parent or partner [27].

Maturity and personality are highlighted as prominent factors in remembering to follow prescribed supplementation. Maturity is a factor that has been shown to influence decision-making in adolescents with type 1 diabetes [30]. Crucial factors in being able to make decision-making in a medical context are communicating a choice, understanding, reasoning, and appreciation. In favourable environmental circumstances, this may be possible at the onset of adolescence; however, the reward system of the brain develops early, and the development of the control system occurs late, which reduces the capacity for decision competence for adolescents in specific situations [31]. From this, it can be concluded that even adolescents with high capacity need support in their decision-making, which emerges as favourable in the results. Several of those who described that they have strategies for their supplementation emphasized the importance of relatives or other important people supporting and reminding them. This is in line with earlier findings that have shown that support from parents and partners can have a positive effect on adherence [32]. Enhanced adherence ought to be achieved if parental and partner involvement (referent power [27]) is included in care to strengthen external motivation.

A distrust of treatment or healthcare providers is known to lower adherence [33], which may come from a lack of health literacy [33, 34] or participants receiving conflicting information from different healthcare providers, the media, or the Internet [35]. Some participants in this study expressed a distrust of the supplements and suggested that it is more effective and natural to eat a varied diet with a lot of vegetables. However, this highlights the need for healthcare providers who have knowledge of this specific group of patients to provide consistent information [36]. Due to the high prevalence of non-adherence to supplementation recommendations after bariatric surgery, healthcare providers should take time to discuss adherence with a focus on problem-solving and different predictors for each individual in an effort to improve adherence.

Our participants described knowledge and understanding as being of importance to adherence, which is in line with a recently published review that pointed out the value of providing clear and consistent information to patients about postoperative lifestyle behaviours [13]. The long-term effectiveness of real-time medication monitoring combined with text message reminders has been shown to improve adherence in adults with type 2 diabetes [37]; however, several educational interventions have only shown modest success in improving adherence to treatment recommendations across chronic diseases [38].

Previous research describes two main barriers for adherence to supplementation recommendations after bariatric surgery: difficulty in swallowing pills and forgetting to take supplements. This is consistent with our results [9, 10]. Taking supplements at a set time each day, keeping them in a visible place, using pill organizers, and/or setting an alarm were some of the factors recommended by participants that helped them to remember to take their supplements. They described better adherence if there is a daily routine which is in line with a recent study [33]. In summary, they are well aware that they have a choice and can act intentionally.

Cost was mentioned by our participants as a barrier due to participants having to pay for the supplements themselves. Thus, an additional approach could be to balance the financial cost against the cost of nutritional deficiencies and to educate patients about the risk of not taking supplementation. Thus, studies have shown that even when medicine is free or co-pays are decreased, lowering costs has only a small influence on improving adherence [39].

Barrett describes power as “the capacity to participate knowingly in change” (awareness, choices, freedom to act intentionally, and involvement in creating change) [29, p 48–49], and in our study, most of the participants have an awareness of that they ought to take their supplementation and which barriers they are experiencing, as well as facilitators. Our study clearly shows that participants are aware that it is they themselves who are creating the change.

This study has several potential limitations including the quantitative cross-sectional design with a small sample size that does not permit a casual interpretation of results. The classification of the direct method measuring adherence may be discussed, but our clinical experiences support the different categories of adherence. Uncontrolled factors such as reduced bioavailability related to obesity, impaired absorption after surgery, and preoperative deficiencies may have influenced our results.

In the qualitative analysis, the authors have strived to obtain trustworthiness (credibility, confirmability, dependability, and transferability). The participants and context are described in as much detail as possible, all authors have been involved in the analysis, and the analysis process is described in as much detail as possible. Furthermore, quotations have been used in the results to strengthen trustworthiness [40]. One strength of the qualitative part of the study is that all participants in the AMOS study from the greater Stockholm area were asked to participate, which resulted in a 50% acceptance rate. For the interview study, nearly 50% of adolescents who had undergone bariatric surgery in the Stockholm region participated in the interview study, which strengthens trustworthiness. However, it is a weakness that they primarily come from urban areas, and this may weaken transferability.

Further studies are needed to understand in more depth the importance of young adults’ awareness and capacity to participate in change. It would be of importance to explore whether the involvement of important people in their lives would affect their ability to adhere to recommendations.

## Conclusion

Bariatric surgery is a comprehensive procedure that requires lifelong treatment afterwards. There is coherence between what adolescents actually do and what they say they do. Capacity is crucial for adherence, and social support has been shown to be important.

## References

[1] Ricci C, Gaeta M, Rausa E, Asti E, Bandera F, Bonavina L (2015). Long-term effects of bariatric surgery on type II diabetes, hypertension and hyperlipidemia: a meta-analysis and meta-regression study with 5-year follow-up. Obes Surg.

[2] Chakhtoura MT, Nakhoul NN, Shawwa K, Mantzoros C, El Hajj Fuleihan GA (2016). Hypovitaminosis D in bariatric surgery: a systematic review of observational studies. Metabolism.

[3] Xanthakos SA, Khoury JC, Inge TH, Jenkins TM, Modi AC, Michalsky MP, Chen MK, Courcoulas AP, Harmon CM, Brandt ML (2020). Nutritional risks in adolescents after bariatric surgery. Clin Gastroenterol Hepatol.

[4] Inge TH, Courcoulas AP, Jenkins TM, Michalsky MP, Brandt ML, Xanthakos SA, Dixon JB, Harmon CM, Chen MK, Xie C, Evans ME, Helmrath MA, Teen–LABS Consortium (2019). Five-year outcomes of gastric bypass in adolescents as compared with adults. N Engl J Med.

[5] De Jong MMC, Hinnen C (2017). Bariatric surgery in young adults: a multicenter study into weight loss, dietary adherence, and quality of life. Surg Obes Relat Dis.

[6] Mechanick JI, Apovian C, Brethauer S, Garvey WT, Joffe AM, Kim J, Kushner RF, Lindquist R, Pessah-Pollack R, Seger J (2019). Clinical practice guidelines for the perioperative nutrition, metabolic, and nonsurgical support of patients undergoing bariatric procedures - 2019 update: cosponsored by American Association of Clinical Endocrinologists/American College of Endocrinology, The Obesity Society, American Society for Metabolic & Bariatric Surgery, Obesity Medicine Association, and American Society of Anesthesiologists - executive summary. Endocr Pract.

[7] Stein J, Stier C, Raab H, Weiner R (2014). Review article: the nutritional and pharmacological consequences of obesity surgery. Aliment Pharmacol Ther.

[8] De Geest S, Sabate E (2003). Adherence to long-term therapies: evidence for action. Eur J Cardiovasc Nurs.

[9] Mahawar KK, Clare K, O'Kane M, Graham Y, Callejas-Diaz L, Carr WRJ (2019). Patient perspectives on adherence with micronutrient supplementation after bariatric surgery. Obes Surg.

[10] Modi AC, Zeller MH, Xanthakos SA, Jenkins TM, Inge TH (2013). Adherence to vitamin supplementation following adolescent bariatric surgery. Obesity (Silver Spring).

[11] Sunil S, Santiago VA, Gougeon L, Warwick K, Okrainec A, Hawa R, Sockalingam S (2017). Predictors of vitamin adherence after bariatric surgery. Obes Surg.

[12] Taddeo D, Egedy M, Frappier JY (2008). Adherence to treatment in adolescents. Paediatr Child Health.

[13] Hood MM, Kelly MC, Feig EH, Webb V, Bradley LE, Corsica J (2018). Measurement of adherence in bariatric surgery: a systematic review. Surg Obes Relat Dis.

[14] Hood MM, Corsica J, Bradley L, Wilson R, Chirinos DA, Vivo A (2016). Managing severe obesity: understanding and improving treatment adherence in bariatric surgery. J Behav Med.

[15] Bergh I, Lundin Kvalem I, Risstad H, Sniehotta FF (2016). Preoperative predictors of adherence to dietary and physical activity recommendations and weight loss one year after surgery. Surg Obes Relat Dis.

[16] Via MA, Mechanick JI (2017). Nutritional and micronutrient care of bariatric surgery patients: current evidence update. Curr Obes Rep.

[17] Olbers T, Beamish AJ, Gronowitz E, Flodmark CE, Dahlgren J, Bruze G, Ekbom K, Friberg P, Gothberg G, Jarvholm K (2017). Laparoscopic Roux-en-Y gastric bypass in adolescents with severe obesity (AMOS): a prospective, 5-year, Swedish nationwide study. Lancet Diabetes Endocrinol.

[18] Parrott J, Frank L, Rabena R, Craggs-Dino L, Isom KA, Greiman L (2017). American Society for Metabolic and Bariatric Surgery Integrated Health Nutritional Guidelines for the Surgical Weight Loss Patient 2016 Update: Micronutrients. Surg Obes Relat Dis.

[19] Karlberg J, Luo ZC, Albertsson-Wikland K (2001). Body mass index reference values (mean and SD) for Swedish children. Acta Paediatr.

[20] Kvale S, Brinkmann S (2009). InterViews : learning the craft of qualitative research interviewing.

[21] Price B (2002). Laddered questions and qualitative data research interviews. J Adv Nurs.

[22] Ross AC, Manson JE, Abrams SA, Aloia JF, Brannon PM, Clinton SK, Durazo-Arvizu RA, Gallagher JC, Gallo RL, Jones G (2011). The 2011 report on dietary reference intakes for calcium and vitamin D from the Institute of Medicine: what clinicians need to know. J Clin Endocrinol Metab.

[23] Krippendorff K (2018). Content analysis : an introduction to its methodology.

[24] Patton MQ (2015). Qualitative research & evaluation methods.

[25] Stirratt MJ, Dunbar-Jacob J, Crane HM, Simoni JM, Czajkowski S, Hilliard ME, Aikens JE, Hunter CM, Velligan DI, Huntley K, Ogedegbe G, Rand CS, Schron E, Nilsen WJ (2015). Self-report measures of medication adherence behavior: recommendations on optimal use. Transl Behav Med.

[26] Cooper PL, Brearley LK, Jamieson AC, Ball MJ (1999). Nutritional consequences of modified vertical gastroplasty in obese subjects. Int J Obes Relat Metab Disord.

[27] Buchmann WF (1997). Adherence: a matter of self-efficacy and power. J Adv Nurs.

[28] Bandura A (1977). Self-efficacy: toward a unifying theory of behavioral change. Psychol Rev.

[29] Barrett EA (2010). Power as knowing participation in change: what’s new and what’s next. Nurs Sci Q.

[30] Viklund G, Wikblad K (2009). Teenagers’ perceptions of factors affecting decision-making competence in the management of type 1 diabetes. J Clin Nurs.

[31] Grootens-Wiegers P, Hein IM, van den Broek JM, de Vries MC (2017). Medical decision-making in children and adolescents: developmental and neuroscientific aspects. BMC Pediatr.

[32] Kyngas H (2004). Support network of adolescents with chronic disease: adolescents’ perspective. Nurs Health Sci.

[33] Durand H, Casey M, Glynn LG, Hayes P, Murphy AW, Molloy GJ (2020). A qualitative comparison of high and low adherers with apparent treatment-resistant hypertension. Psychol Health Med.

[34] Zhang NJ, Terry A, McHorney CA (2014). Impact of health literacy on medication adherence: a systematic review and meta-analysis. Ann Pharmacother.

[35] Carpenter DM, Elstad EA, Blalock SJ, DeVellis RF (2014). Conflicting medication information: prevalence, sources, and relationship to medication adherence. J Health Commun.

[36] Nordin K, Brorsson AL, Ekbom K (2018). Adolescents’ experiences of obesity surgery: a qualitative study. Surg Obes Relat Dis.

[37] Vervloet M, van Dijk L, de Bakker DH, Souverein PC, Santen-Reestman J, van Vlijmen B, van Aarle MC, van der Hoek LS, Bouvy ML (2014). Short- and long-term effects of real-time medication monitoring with short message service (SMS) reminders for missed doses on the refill adherence of people with type 2 diabetes: evidence from a randomized controlled trial. Diabet Med.

[38] Kini V, Ho PM (2018). Interventions to improve medication adherence: a review. JAMA.

[39] Brown MT, Bussell J, Dutta S, Davis K, Strong S, Mathew S (2016). Medication adherence: truth and consequences. Am J Med Sci.

[40] Polit DF, Beck CT (2016). Nursing research : generating and assessing evidence for nursing practice.

